# Emergence of melioidosis in the Indian Ocean region: Two new cases and a literature review

**DOI:** 10.1371/journal.pntd.0006018

**Published:** 2017-12-14

**Authors:** Nicolas Allou, Olivier Martinet, Jérôme Allyn, Bruno Bouchet, Marie-Christine Jaffar-Bandjee, Thomas Galas, Nicolas Traversier, Olivier Belmonte

**Affiliations:** 1 Réanimation Polyvalente, Centre Hospitalier Universitaire Félix Guyon, Saint Denis, France; 2 Bacteriologie, Centre Hospitalier Universitaire Felix Guyon, Saint Denis, France; University of California San Diego School of Medicine, UNITED STATES

## Abstract

Melioidosis is a disease caused by bacteria called *B*. *pseudomallei*. Infections can develop after contact with standing water. This disease can reach all the organs and especially the lungs. It is associated with a high mortality rate (up to 50%). Melioidosis is endemic in northern Australia and in Southeast Asia. Nevertheless, *B*. *pseudomallei* may be endemic in the Indian Ocean region and in Madagascar in particular, so clinicians and microbiologists should consider acute melioidosis as a differential diagnosis in the Indian Ocean region, in particular from Madagascar.

## Overview

Melioidosis is a frequent disease caused by *Burkholderia pseudomallei* and found mostly in northern Australia and Southeast Asia. In the last decade, increasingly more cases of melioidosis have been reported in the Indian Ocean region, particularly in Madagascar. Here, we report two new cases of melioidosis complicated by multiple organ failure that were observed in Réunion Island. The two affected patients came from abroad: one patient—whose diagnosis was delayed by one year—came from Madagascar, and the other came from Southeast Asia.

Our two cases and our literature review suggest the importance of considering the diagnosis of melioidosis in patients returning from the Indian Ocean region, in particular from Madagascar. Further studies are needed in order to investigate the real incidence of melioidosis in the Indian Ocean region (especially in Madagascar) because this incidence may be higher than assumed.

## Introduction

Melioidosis is a disease caused by *B*. *pseudomallei* and found mostly in northern Australia and in Southeast Asia [1, 2]. For the last decade, the number of melioidosis cases in the Indian Ocean region have seemed to be on the rise [3–9], particularly in Madagascar [6–9]. Here, we report two new cases of melioidosis complicated by multiple organ failure that were observed in Réunion Island, which is a French overseas department. We also provide a literature review on melioidosis in the Indian Ocean, a region composed of Madagascar, the Archipelagos of Comoros, the Seychelles, Mauritius, and Réunion Island.

## Cases

### Case summary 1

In 2016, a 63-year-old French man with no past medical history who lived in Madagascar and made frequent trips to Mayotte experienced an infection with cough and fever for one week. The patient was initially treated with ofloxacin. His health worsened with nausea, vomiting, dehydration, and consciousness disorder, and he was transferred from Madagascar to Réunion Island. On arrival, he had septic shock with coma with a Glasgow scale score of 8, prompting orotracheal intubation and transfer to the intensive care unit. The physical examination yielded only hepatomegaly. Blood tests revealed lymphopenia (0.19 G/L), thrombopenia (70 G/L), a prothrombin time of 10%, a blood lactate concentration of 18 mmol/L, and an elevated C-reactive protein of 287 mg/L. A total-body computed tomography (TB-CT) scan showed multiple bilateral lung abscesses, a liver abscess, splenomegaly, and hepatomegaly. Antimicrobial therapy with meropenem (6 g/day), colimycin (9 million international units/day), amikacin, and artesunate was immediately started. The evolution was marked by multiple organ failure, and the patient died on day one. Matrix-assisted laser desorption ionization time-of-flight (MALDI-TOF) mass spectrometry (Bruker Biotyper) identified *B*. *thailandensis* in the patient’s blood cultures and respiratory samples. The strain was susceptible in vitro to ticarcillin/clavulanic acid, trimethoprim/sulfamethoxazole, and ceftazidime, and it was resistant to meropenem and levofloxacin. The patient died with a preliminary diagnosis of *B*. *thailandensis* pneumonia. One year later, after we diagnosed our second case of melioidosis (see below), we tested the dead patient’s strain with real-time PCR assays using hydrolysis probes designed for three genetic markers of the type III secretion system (*orf11*, *orf13*, and *BpSCU2*). This led to the identification of *B*. *pseudomallei*. It turned out that the isolate had initially been misidentified as *B*. *thailandensis* because *B*. *pseudomallei* was not included in the MALDI-TOF database.

### Case summary 2

In 2017, a 40-year-old man who lived in Indonesia and had no past medical history made stops in Southeast China and Singapore while working on a cruise ship as a fitter’s mate. Fifteen days after departure from Singapore to Indonesia, he presented a fever and was treated with amoxicillin/clavulanic acid. Five days later, his health worsened. He was transferred by helicopter from the ship to Réunion Island. On arrival, he had septic shock with multiple organ failure, leading to orotracheal intubation and transfer to the intensive care unit. On admission, he presented a fever (40.6°C), and the physical examination showed hepatomegaly and extensive skin pustules on the face. Blood tests revealed lymphopenia (0.53 G/L), thrombopenia (29 G/L), an elevated serum procalcitonin level (72.8 μg/L), and a blood lactate concentration of 3.3 mmol/L. A TB-CT scan showed cerebral venous thrombosis, multiple bilateral lung abscesses, a liver abscess, splenomegaly, and hepatomegaly. Antimicrobial therapy with high doses of meropenem and amikacin was immediately started. MALDI-TOF found *B*. *thailandensis* in the patient’s blood cultures, respiratory samples, and skin abscess. Real-time PCR assays with hydrolysis probes designed for three genetic markers of type III secretion system (*orf11*, *orf13*, and *BpSCU2*) identified *B*. *pseudomallei*. The strain was susceptible in vitro to ticarcillin/clavulanic acid (minimal inhibitory concentration [MIC] of 3 mg/L), meropenem (MIC of 0.5 mg/L), ceftazidime (MIC of 1 mg/L), and tetracycline (MIC of 1.5 mg/L). The strain was resistant to trimethoprim/sulfamethoxazole and levofloxacin. The evolution was marked by microbiological failure, and all of the daily blood cultures remained positive until day 10 despite treatment with continuous infusion of meropenem (6 g/day). In view of this, a second TB-CT scan and a magnetic resonance imaging of the brain were performed, which showed two subdural empyemas (of 9.5 mm). Antibiotic treatment was changed to ceftazidime (12 g/day with continuous infusion). The patient’s condition improved, and he was discharged from the intensive care unit on day 15 and on day 70, respectively. The last TB-CT scan and magnetic resonance imaging of the brain were performed seven days before the patient was discharged from the hospital and showed a reduction in the size of the bilateral lung abscesses, an almost complete regression of splenic and hepatic abscesses, and a significant reduction of the cerebral abscesses.

## Literature review and discussion

The first human case of melioidosis in the Indian Ocean region was observed in Réunion Island in 2004 in a patient who came from Madagascar [6]. Our review of the literature shows that 13 cases of melioidosis have been documented in the Indian Ocean region since 2004, including our two cases (Table 1) [3–9].

**Table 1:** Characteristics of the patients with melioidosis in Indian Ocean region.


Case [reference]	Year	Area of diagnosis	Country visited last year	Age	Sex	Comorbidity	Presenting feature	Preliminary identification	Delay of diagnosis	Technique of identification of *B*. *pseudomallei*	Antibiotic therapy	Duration of treatment	Death
1 [6]	2004	Réunion Island	Madagascar	60	male	heavy alcohol use	pneumonia, bacteremia	*Pseudomonas aeruginosa*	5 days	phenotypic (API-20NE system)	ceftazidime/trimethoprim—sulfamethoxazole	20 weeks	no
2 [6]	2004	Réunion Island*	0	81	male	chronic pulmonary disease	pneumonia, bacteremia	-	0	phenotypic (API-20NE system)	ceftazidime/trimethoprim—sulfamethoxazole	20 weeks	no
3 [6]	2004	Réunion Island*	0	xx	female	glucocorticoid therapy	pneumonia, bacteremia	-	0	phenotypic (API-20NE system)	ceftazidime/trimethoprim—sulfamethoxazole	20 weeks	no
4 [3]	2004	Mauritius	0	40	female	glucocorticoid therapy	skin and soft tissue infection, bacteremia	-	14 days	phenotypic (API-20NE system)	ceftazidime	9 days	yes
5 [8]	2005	Réunion Island	Madagascar	58	male	no	pneumonia	-	20 days	phenotypic (API-20NE system) then real-time PCR	imipenem/trimethoprim—sulfamethoxazole/doxycycline	23 weeks	no
6 [4]	2012	Réunion Island	0	57	male	no	periprostatic abscesses, bacteremia	-	2 days	phenotypic (Vitek 2 Compact) then real-time PCR	ceftazidime/doxycycline	32 weeks	no
7 [7]	2012	Madagascar	0	52	male	no	pneumonia, splenic and liver abscesses, bacteremia	-	12 days	phenotypic (API-20NE system)	ceftriaxone	3 days	yes
8 [7]	2013	Madagascar	0	45	male	diabetes	splenic and liver abscesses, bacteremia	-	16 days	phenotypic (API-20NE system) then real-time PCR	ceftazidime	24 hours	yes
9 [9]	2013	Denmark	Madagascar	43	male	no	pneumonia, periprostatic abscesses, spondylitis	*Burkholderia cepacia*	20 days	phenotypic (Vitek 2 Compact) then MALDI-TOF mass spectrometry	meropenem/trimethoprim—sulfamethoxazole	unknown	no
10 [5]	2013	Seychelles**	0	xx	male	heavy alcohol use	pneumonia	-	Weeks	real-time PCR	unknown	unknown	yes
11 [5]	2013	Seychelles**	Mauritius	xx	male	heavy alcohol use	pneumonia	-	Weeks	phenotypic (Vitek 2 Compact) then real-time PCR	unknown	unknown	no
12	2016	Réunion Island	Madagascar, Mayotte	63	male	no	pneumonia, splenic and liver abscesses, bacteremia	*B*. *thailandensis*	24 months	real-time PCR targeting type III secretion system genes	meropenem	24 hours	yes
13	2017	Réunion Island	Southeast Asia	40	male	no	pneumonia, skin lesions, splenic and liver abscesses, bacteremia	*B*. *thailandensis*	2 days	real-time PCR targeting type III secretion system genes	ceftazidime/doxycycline	34 weeks	no


*Case 2 and case 3 were two nosocomial cases with the same strain as case 1.

**Case 10 and case 11 worked in the same place.

Abbreviations: API, analytical profile index; MALDI-TOF, matrix-assisted laser desorption ionization time-of-flight.

The majority of these cases occurred after a stay in Madagascar (46.2%) [6,9]. Most of the patients were male, and seven had an underlying disease (53.8%). Pneumonia was reported in nine cases (69.2%), and bacteremia was present in eight cases (61.5%). Diagnosis was delayed in almost all of the cases (Table 1), even until after death for four patients (30.7%; cases 7, 8, 10, and 12), which probably led to death in these cases. Microorganisms were initially misidentified by an automated microbiology system in four cases (30.7%).

In the Indian Ocean region, most of the reported cases of melioidosis occurred after a stay in Madagascar [6,9]. However, the diagnosis of melioidosis is made more frequently outside of the country [6,8,9]. This is because melioidosis is difficult to diagnose in Madagascar, where laboratories are not equipped to identify such microorganisms. The incidence of melioidosis in Madagascar may be more important than assumed because it is likely that many cases are never diagnosed or treated. Even in countries with high medical standards, the diagnosis of melioidosis can be missed because physicians in nonendemic regions lack knowledge of this disease. Moreover, *B*. *pseudomallei* can be misidentified by automated microbiology systems such as MALDI-TOF, as et alillustrated by case 1 of this study as well as by the case described by Weissert. in the context of Switzerland [10]. In clinical practice, the misidentification by MALDI-TOF could have consequences for the antibiotic that is selected. After recovery of the antibiogram, clinicians may administer a treatment to which the isolated microorganism is interpreted as sensitive but which is not the reference treatment for melioidosis [11].

When analyzing uncommon pathogenic microorganisms such as *Burkholderia* spp. in patients with atypical clinical presentations or in patients returning from a region where melioidosis is present, microbiologists and clinicians should take into account the risk of misidentification by MALDI-TOF. Complementary testing should be performed on these microorganisms with conventional biochemical analyses (analytical profile index [API]-20NE system or Vitek 2 Compact) or at best with molecular identification procedures such as 16S rRNA gene sequence analysis or with real-time PCR assays targeting genetic markers.

Nosocomial risk of melioidosis in French overseas territories like Réunion Island needs to be carefully monitored. The high incidences of diabetes and climatologic conditions such as rainy seasons with the occurrence of tropical cyclones make Réunion Island a possible setting for melioidosis. Moreover, in Réunion Island, an autochthonous case of melioidosis had recently been reported [4], and two nosocomial cases had occurred a few years ago [6] in a region where clinicians and microbiologists are not accustomed to this disease. Furthermore, the majority of melioidosis cases in the Indian Ocean region had a link with Madagascar, a country close to Réunion Island where many patients are repatriated each year (500 between 2010 and 2015) [12].

The mode of contamination, which occurred during the rainy season in Réunion Island, was difficult to explain for case 6 (Table 1). It seems very unlikely that our patient acquired the infection outside of Réunion Island. The only foreign country the patient had visited was Mauritius five years earlier, where only one case of melioidosis has been described to date [3]. Moreover, considering the acute presentation of this case 6, an incubation period of five years seems unlikely even if the longest reported incubation period was 62 years [13].

An interesting point of our case report number 2 (Table 1) is the cutaneous manifestation of melioidosis, which was a secondary skin melioidosis with multiple pustules from hematogenous spread. This cutaneous form of melioidosis is rare (2% in the study by Katherine et al.) [14] and is different from primary skin melioidosis, which is more frequent (>10%) and usually occurs at the site of inoculation. Moreover, primary skin melioidosis is associated with a better outcome and with fewer risk factors than other forms of melioidosis [14].

## Conclusions

Overall, clinicians and microbiologists should be aware of the risk of melioidosis in travelers returning from the Indian Ocean region (especially from Madagascar) for the following reasons: (1) melioidosis is a severe disease responsible for community-acquired infections with strains resistant to many common broad-spectrum antibiotics, (2) diagnosis of melioidosis in nonendemic regions is frequently delayed, (3) early administration of effective antibiotic therapy has been shown to have a beneficial effect on outcome in patients with melioidosis [11], and (4) *B*. *pseudomallei* may be endemic in the Indian Ocean region and in Madagascar in particular.

**Key learning points**

- *B*. *pseudomallei* may be endemic in the Indian Ocean region and in Madagascar in particular.
- Clinicians and microbiologists should consider acute melioidosis as a differential diagnosis in the Indian Ocean region, in particular in Madagascar.
